# Nitrogen Addition Exacerbates the Negative Effects of Low Temperature Stress on Carbon and Nitrogen Metabolism in Moss

**DOI:** 10.3389/fpls.2017.01328

**Published:** 2017-08-02

**Authors:** Bin-Yang Liu, Chun-Yi Lei, Wei-Qiu Liu

**Affiliations:** ^1^Guangdong Key Laboratory of Plant Resources, School of Life Sciences, Sun Yat-sen University Guangzhou, China; ^2^State Key Laboratory of Vegetation and Environment Change, Institute of Botany, Chinese Academy of Sciences Beijing, China; ^3^Department of Scientific Research and Education, Heishiding Nature Reserve Zhaoqing, China

**Keywords:** abiotic stress, compensation effect, low temperature, nitrogen deposition, carbon metabolism, nitrogen metabolism, moss

## Abstract

Global environmental changes are leading to an increase in localized abnormally low temperatures and increasing nitrogen (N) deposition is a phenomenon recognized worldwide. Both low temperature stress (LTS) and excess N induce oxidative stress in plants, and excess N also reduces their resistance to LTS. Mosses are primitive plants that are generally more sensitive to alterations in environmental factors than vascular species. To study the combined effects of N deposition and LTS on carbon (C) and N metabolism in moss, two moss species, *Pogonatum cirratum* subsp. *fuscatum*, and *Hypnum plumaeforme*, exposed to various concentrations of nitrate (KNO_3_) or ammonium (NH_4_Cl), were treated with or without LTS. C/N metabolism indices were then monitored, both immediately after the stress and after a short recovery period (10 days). LTS decreased the photosystem II (PSII) performance index and inhibited non-cyclic photophosphorylation, ribulose-1,5-bisphosphate carboxylase, and glutamine synthetase activities, indicating damage to PSII and reductions in C/N assimilation in these mosses. LTS did not affect cyclic photophosphorylation, sucrose synthase, sucrose-phosphate synthase, and NADP-isocitrate dehydrogenase activities, suggesting a certain level of energy and C skeleton generation were maintained in the mosses to combat LTS; however, LTS inhibited the activity of glycolate oxidase. As predicted, N supply increased the sensitivity of the mosses to LTS, resulting in greater damage to PSII and a sharper decrease in C/N assimilation. After the recovery period, the performance of PSII and C/N metabolism, which were inhibited by LTS increased significantly, and were generally higher than those of control samples not exposed to LTS, suggesting overcompensation effects; however, N application reduced the extent of compensation effects. Both C and N metabolism exhibited stronger compensation effects in *H. plumaeforme* than in *P. cirratum* subsp. *fuscatum*. The difference was especially pronounced after addition of N, indicating that *H. plumaeforme* may be more resilient to temperature and N variation, which could explain its wider distribution in the natural environment.

## Introduction

The overall trend of global climate change is warming; however, the accelerated melting and disappearance of Arctic Sea-Ice has also led to increasing local occurrences of abnormally low temperatures (Mori et al., 2014; Overland et al., 2015). Low temperature stress (LTS) damages plants by inducing oxidative stress, hindering photosynthesis, and indirectly inducing osmotic stress (Nuccio et al., 1999; Allen and Ort, 2001; Beck et al., 2007). Increasing nitrogen (N) deposition is another environmental problem which attracts global attention (Stevens et al., 2004; Galloway et al., 2008; Liu et al., 2013). Excess N deposition leads to nutritional imbalance, cell membrane destruction, and oxidative stress in plants (Pearce and van der Wal, 2002; Koranda et al., 2007). N in wet deposition mainly occurs in the forms of ${\text{NO}}_{3}^{-}$ and ${\text{NH}}_{4}^{+}$, but they have different effects on plants. On the one hand, as H^+^ is generated during ${\text{NH}}_{4}^{+}$ assimilation, whereas OH^−^ is generated during ${\text{NO}}_{3}^{-}$ assimilation, ${\text{NH}}_{4}^{+}$ is more apt to decrease tissue pH and disrupt membrane function (Pearce et al., 2003; Paulissen et al., 2004). On the other hand, ${\text{NH}}_{4}^{+}$ is more readily available than ${\text{NO}}_{3}^{-}$ for utilization by plants as less energy is needed during its assimilation (Flaig and Mohr, 1992). Moreover, excess N deposition can reduce the resistance of plants to LTS (Fenn et al., 1998); this phenomenon merits attention, although the underlying mechanisms have seldom been investigated.

The resilience of plants to stress, namely the ability of plants to recover growth after removal of stress factors, is vital for their survival in natural environments. This phenomenon, referred to as the “compensation effect” (Belsky, 1986), exists widely in various plants, and has been studied extensively in agriculture and stockbreeding. However, the effects of N deposition on compensation effects after LTS in plants is poorly understood, despite the importance of precise evaluation of the effects of global change on plants and ecosystems.

Mosses, a group of primitive land plants with small stature that mainly acquire their nutrients from air, are relatively sensitive to environmental change, and, therefore, generally more vulnerable to global changes than vascular plants (Pearce and van der Wal, 2002; Harmens et al., 2011; Schröder et al., 2014). Comprehensive understanding of the stress responses of mosses and the mechanisms underlying them will facilitate accurate evaluation of the fate of mosses on the background of global change. Studies of the responses of mosses to LTS have primarily been limited to the model species Physcomitrella patens (Nagao et al., 2006; Bhyan et al., 2012), and little attention has been paid to other species or their ecological roles (Yin and Zhang, 2016). Moreover, there are no reports detailing the responses of mosses to combined high N and LTS, or their recovery characteristics.

*Pogonatum cirratum* Brid. subsp. *fuscatum* (Mitt.) Hyvönen (hereafter referred to as *P. cirratum*) and *Hypnum plumaeforme* Wilson, are both common moss species in China, with *H. plumaeforme* the more widely distributed organism. These plants are suitable for the study of stress responses, as previously described (Liu et al., 2015). We examined their primary metabolism, secondary metabolism, and hormone regulation responses to LTS, as well as their recovery characteristics in response to addition of various N concentrations (KNO_3_ or NH_4_Cl). The results of analysis of secondary metabolism have previously been reported (Liu et al., 2015). In the present study, we focused on responses involving photosynthesis, inorganic C and N assimilation, and amino acid metabolism. The aims of the study were to: (1) comprehensively analyze the effects of combined high N and LTS on C and N metabolism in moss, including interactions between metabolic pathways; (2) explore compensation effects in moss after exposure to these stresses and their underlying mechanisms; (3) compare the resilience of the two tested species to LTS and N stress.

## Materials and methods

### Experimental design and stress treatment

The study was conducted at Heishiding Nature Reserve (23°27′ N, 111°53′ E) in Guangdong Province, southern China. The Reserve is located in a subtropical moist forest zone and has a monsoon climate. The mean annual temperature is 19.6°C, with average temperatures during the coldest (January) and hottest (July) months of 10.6 and 28.4°C, respectively. The mean annual rainfall is 1743.8 mm, with 79% of it falling from April to September. The soils in the study sites are lateritic red earth. Annual wet N deposition in the area has been recorded as 9.6–10.0 kg ${\text{NH}}_{4}^{+}$-N hm^−2^ and 6.5-8.1 kg ${\text{NO}}_{3}^{-}$-N hm^−2^ (Fang et al., 2011).

Two moss species, *P. cirratum* and *H. plumaeforme* were subjected to nitrogen and low-temperature treatment as previously described (Liu et al., 2015). In brief, after an acclimation of two months in the field, the mosses were exposed to nitrate (KNO_3_) or ammonium (NH_4_Cl) application at three rates (20, 40, and 60 kg N hm^−2^, representing low, medium and high N application, respectively); controls were treated with distilled water. N-treated and control groups were designated +N and −N, respectively. After the final N or water application, +N and −N trays containing each species were exposed to low-temperature [1°C (dark)/3°C (light)] or control [7°C (dark)/15°C (light)] treatments, designated +LTS and −LTS respectively. After 10 days of treatment, half of the moss samples in each tray were collected for physiological measurements, and then the trays containing the remaining mosses were carried back to the field for a recovery period of 10 days. After recovery, the remaining samples were collected for measurements. Post-recovery −N/−LTS, +N/−LTS, −N/+LTS, and +N/+LTS samples were designated R.−N/−LTS, R.+N/−LTS, R.−N/+LTS, and R.+N/+LTS, respectively.

### Physiological measurements

#### Chlorophyll fluorescence

Chlorophyll fluorescence was determined using a Plant Efficiency Analyzer (PEA, Hansatech Ltd., UK). Immediately after fresh samples were cut from the top 2 cm of individual plants and attached to each leaf clip with one shoot, they were dark adapted for 20 min, and then the maximum fluorescence intensity (F_M_), and the fluorescence intensities at 0 s (F_0_), 300 μs (F_300_ μs), and 2 ms (F_J_) were determined with an excitation light intensity of 3,000 mmol m^−2^s^−1^. The behavior of photosystem II (PSII) was quantified by the performance index on absorption basis (PIabs), which is an overall expression indicating a kind of internal force of the resist constraints from outside, it was calculated as shown in Equation (1) (Strasser and Strasser, 1995; Appenroth et al., 2001).

(1) $$Plabs=\frac{{\left(F_{M}-F_{0}\right)}^{2}}{\left[4F_{M}\times F_{0}\times (F_{300\;\mu \text{s}}-F_{0})\right]}\times (F_{M}-F_{J})$$

#### Enzyme activities

Enzymes were extracted and chloroplast suspensions prepared using the methods described by Liu et al. (2016). Briefly, 1.5 g fresh sample was ground in liquid nitrogen and then added to 8 ml ice-cold Tris-HCl buffer (50 mmol l^−1^, pH 7.5) and 0.1 ml phenylmethanesulfonyl fluoride-isopropanol solution (0.4 mol l^−1^). The mixture was centrifuged (7,000 × g, 4°C, 4 min) and the supernatant used for determination of enzyme activity, while the pellet was resuspended in 4 ml ice-cold Tris-HCl buffer (50 mmol l^−1^, pH 7.5) and the resulting chloroplast suspension used for determination of photophosphorylation activity.

CPSP (cyclic photophosphorylation) and NCPSP (non-cyclic photophosphorylation) activity was calculated by the consuming of inorganic phosphorus, and represented by the synthesis of ATP (SIPP, 1999). In brief, One ml of the chloroplast suspension was added into 1 ml of 0.05 M Tris-HCl buffer (pH 7.5) containing 1 mM MgCl_2_, 0.5 mM Na_2_HPO_4_, 0.5 mM ADP-Na_2_, 0.03 mM 5-methylphenazonium methosulfate (for CPSP)/0.5 mM NADP-Na and 0.5 mM K_3_Fe(CN)_6_ (for NCPSP). After 30 min of illumination (60 μmol m^−2^ s^−1^, at room temperature), 1 ml of 20% trichloroacetic acid was added to halt the reaction. The reaction buffer was centrifuged at 12,000 rpm for 2 min, and 2.5 ml of the coloration buffer [containing 6% FeSO_4_, 3% (NH_4_)_6_Mo_7_O_24_ and 1.5 M H_2_SO_4_] was added into 0.5 ml of the supernatant, then the absorbance was quantified spectrophotometrically at 660 nm to detect the inorganic phosphorus content. An ultraviolet spectrophotometer (WFZ UV-2000, UNICO Ltd., Shanghai, China) was used for all colorimetric measurements in this study.

Ribulose-1,5-bisphosphate carboxylase (RuBPC, E.C. 4.1.1.39) was determined according to SIPP (1999). Eighty microliters enzyme extract was added to 0.5 ml 50 mM Tris-HCl buffer (pH 8.2) containing 10 mM MgCl_2_, 0.6 mM dithiothreitol, 14 mM NaHCO_3_, and 0.4 mM NADH, after equilibrated at 25°C for 5 min, solutions of 70 μl 6 mM ATP, 70 μl 6 mM creatine phosphate sodium, 35 μl 160 U ml^−1^ phosphocreatine kinase, 35 μl 160 U ml^−1^ 3-phosphoglycerate kinase, and 35 μl 160 U ml^−1^ glyceraldehyde-3-phosphate dehydrogenase was added successively. The reaction was initiated by adding 35 μl 9 mM RuBP and then, the absorbance of the mixture was measured spectrophotometrically at 340 nm every minute for 3 min. The enzyme activity was calculated by the consumption of NADH per min (SIPP, 1999). As NADH levels remained stable in the reaction system without adding RuBP, creatine and related enzymes, with its consumption rate being lower than 1% (for detail c.f. Figure S1), the self-decomposition of NADH was ignored in calculation of RuBPC activity.

Glycolate oxidase (GO, E.C. 1.1.3.15) activity was measured according to SIPP (1999). A portion of 0.4 ml enzyme reaction was adding to 0.7 ml 0.1 M PBS (pH 8.0) buffer, and then 0.1 ml 0.7 mM flavin mononucleotide and 0.1 ml 35 mM phenylhydrazine hydrochloride was added. After equilibrated at 30°C for 10 min, 0.1 ml 35 mM sodium glycollate was added to initiate the reaction. After 10 min of reaction, 0.1 ml of 2 M HCl was added to halt the reaction, and 1 ml of concentrated hydrochloric acid and 0.2 ml of 0.05 M K_3_Fe(CN)_6_ were added to the buffer, then the absorbance at 550 nm was measured spectrophotometrically after 20 min.

Sucrose phosphate synthase (SPS, E.C. 2.4.1.14) activity was measured according to SIPP (1999). A portion of 0.1 ml enzyme extract was added to 0.15 ml 50 mM Tris-HCl buffer (pH 7.0) containing 10 mM MgCl_2_, 7 mM uridine diphosphate glucose and 10 mM D-fructose 6-phosphate disodium salt, and was incubated at 30°C for 30 min, then mixed with 0.05 ml 2 M NaOH and boiled for 10 min. After cooling, the mixture was added to 0.7 ml 30% HCl and 0.2 ml 0.1% resorcinol-alcohol solution, and incubated at 80°C for 10 min, then analyzed spectrophometrically at 480 nm after cooling. The activity was represented by the generation of sucrose.

Sucrose synthase (SS, E.C. 2.4.1.13) activity was measured according to SIPP (1999). The measurement process was same as SPS except that the buffer contained 10 mM fructose instead of 10 mM D-fructose 6-phosphate disodium salt.

Glutamine synthetase (GS, E.C. 2.7.7.42) activity was measured according to a modified version of the SIPP (1999) method. A portion of 0.6 ml 50 mM Tris-HCl buffer (pH 7.5) containing 0.25 M Glu-Na and 0.15 M NH_4_Cl was mixed with 0.1 ml 0.1 M ATP, 0.1 ml 1 M MgSO_4_, and 0.4 ml enzyme extract, and incubated at 37°C for 30 min, then 0.8 ml of 20% trichloroacetic acid was added to halt the reaction and the mixture was centrifuged at 10,000 rpm for 5 min. One milliliter of the supernatant was mixed with 2.5 ml coloration buffer [containing 6% FeSO_4_, 3% (NH_4_)_6_Mo_7_O_24_ and 1.5 M H_2_SO_4_], the inorganic phosphorus content was analyzed by phosphorus molybdenum blue spectrophotometry at 660 nm (GB/T 9727-2007, 2007). The control was treated with the same reagents and conditions except that the enzyme extract was added after the trichloroacetic acid. The enzyme activity was defined as the difference between the reaction and the control and represented as the ATP consumed per min.

Glutamate dehydrogenase (GDH, E.C. 1.4.1.2) was measured using a modified version of the Moyano et al. (1995) method. A portion of 0.86 ml 50 mM Tris-HCl reaction buffer (pH 8.2) containing 50 mM α-ketoglutaric acid and 40 mM NH_4_Cl was mixed with 75 μl enzyme extract and incubated at 30°C for 2 min. Then, 65 μl 10 mM NADPH solution was added to initiate the reaction. Absorbance at 340 nm was measured spectrophotometrically immediately, and then measured once per minute for 3 min. The enzyme activity was defined as the NADPH consumed per min.

NADP-isocitrate dehydrogenase (IDH, E.C. 1.1.1.42) was measured using a modified version of the colorimetric method of Martínez-Rivas and Vega (1998). In brief, a 0.7 ml aliquot of reaction buffer containing 50 mM Tris-HCl (pH 7.5) and 4.5 mM isocitric acid trisodium salt was added to 0.1 ml of enzyme extract, 0.1 ml 7.4 mM MnCl_2_·2H_2_O, and 0.1 ml 2 mM NADP-Na. The reaction was initiated by adding NADP-Na and enzyme activity defined as NADPH generated per min.

The concentration of soluble protein was measured spectrophotometrically at 595 nm using the protein-Coomassie Brilliant Blue G-250 binding assay. Details of the assay have been provided in Liu et al. (2011).

#### N and free amino acid (FAA) content

N and FAA contents were determined using the method described in Liu et al. (2016). Total N and non-protein nitrogen (NPN) contents were determined by salicylic acid colorimetry (HJ 536-2009, 2009). Protein nitrogen content (PN) was calculated as the difference between total N and NPN. Total FAA, arginine (Arg), and proline (Pro) contents were measured by ninhydrin colorimetry, naphthol colorimetry, and acidic ninhydrin colorimetry, respectively (Troll and Lindsley, 1955; Wang, 2006; He et al., 2007).

### Data analysis

Differences in the indices associated with N treatments under each temperature treatment were analyzed using one-way ANOVA, and the LSD test was used to identify differences significant at the 0.05 probability level. The relationships of each physiological index with N addition levels in the −LTS, +LTS, R. −LTDS, and R.+LTS treatment groups were evaluated by linear regression analysis. A student's *t*-test was used to detect significant differences in physiological indices between temperature treatments (−LTS, +LTS, R.−LTS, and R.+LTS) under the same N conditions or between N forms under the same temperature treatments and N levels (at 0.05 confidence limits).

Recovery rate was determined as the change in physiological indices after recovery and was calculated as the difference in physiological indices between the time after recovery and the time immediately after stress (cf. van Ruijven and Berendse, 2010). And then, one-way ANOVA was used to test significant difference of recovery rate between N addition levels (LSD test was used to identify differences significant at the 0.05 probability level). In addition, one sample-*T*-test was used to test the significant difference between recovery rate and value 0 (a significant positive recovery rate represent an increase, while a significant negative recovery rate represent a decrease in the physiological indices).

All statistical analyses were carried out using SPSS 13.0 software.

## Results

### Chlorophyll fluorescence and photophosphorylation

For −LTS samples, N application decreased PIabs in both species, while it only decreased NCPSP and CPSP in *H. plumaeforme*, and ammonium was associated with much larger declines than nitrate. LTS caused reductions in PIabs of 23 and 32% and in NCPSP of 15 and 29%, in *P. cirratum* and *H. plumaeforme*, respectively. The combination of N application and LTS caused further reductions, ammonium inducing a more pronounced effect. In contrast, LTS caused significant increases in CPSP activity of 100 and 32% in −N samples of *P. cirratum* and *H. plumaeforme*, respectively. N application led to greater inhibition of CPSP in +LTS than −LTS samples, reducing the differences between the corresponding −LTS and +LTS samples (Table 1).

**Table 1:** The performance index (PIabs) of photosystem II, and non-cyclic and cyclic photophosphorylation activities (NCPSP and CPSP, respectively) in *Pogonatum cirratum* subsp. *fuscatum* and *Hypnum plumaeforme* exposed to the indicated amount of N addition, with or without low temperature stress immediately (+LTS and −LTS respectively), or after a 10-day-recovery period (R.+LTS and R.−LTS).

**N treatment (kg N hm^−2^ year^−1^)**		**Nitrate treatment**			**Ammonium treatment**		
		**PIabs**	**NCPSP (μmol ATP mg^−1^chl min^−1^)**	**CPSP (μmol ATP mg^−1^chl min^−1^)**	**PIabs**	**NCPSP (μmol ATP mg^−1^chl min^−1^)**	**CPSP (μmol ATP mg^−1^chl min^−1^)**
***Pogonatum cirratum* subsp. *fuscatum***							
−LTS	0	0.778 ± 0.023c	0.26 ± 0.02 a	0.05 ± 0.01a	0.778 ± 0.023d	0.26 ± 0.02a	0.05 ± 0.01a
	20	0.745 ± 0.010c	0.26 ± 0.02a	0.05 ± 0.01a	0.679 ± 0.024c	0.21 ± 0.01b	0.07 ± 0.02a
	40	0.609 ± 0.016 b	0.25 ± 0.01a	0.05 ± 0.01a	0.502 ± 0.028b	0.22 ± 0.01b	0.05 ± 0.01a
	60	0.507 ± 0.021a	0.24 ± 0.01a	0.05 ± 0.01a	0.329 ± 0.022a	0.16 ± 0.01c	0.04 ± 0.01a
+LTS	0	**0.595 ± 0.021c**	**0.22 ± 0.01b**	**0.10 ± 0.01b**	**0.595 ± 0.021d**	**0.22 ± 0.01c**	**0.10 ± 0.01b**
	20	**0.579 ± 0.016c**	**0.18 ± 0.02ab**	**0.08 ± 0.01a**	**0.493 ± 0.015c**	0.20 ± 0.01c	0.09 ± 0.01ab
	40	**0.421 ± 0.004b**	**0.14 ± 0.01a**	**0.09 ± 0.01ab**	**0.385 ± 0.008b**	**0.15 ± 0.01b**	0.07 ± 0.02a
	60	**0.359 ± 0.017a**	**0.14 ± 0.01a**	0.07 ± 0.01a	0.311 ± 0.011a	**0.12 ± 0.01a**	0.06 ± 0.01a
R.−LTS	0	0.799 ± 0.015b	0.23 ± 0.02a	0.05 ± 0.01a	0.799 ± 0.015d	0.23 ± 0.02b	0.05 ± 0.01b
	20	0.777 ± 0.010b	0.22 ± 0.02a	0.05 ± 0.02a	0.741 ± 0.014c	0.20 ± 0.02ab	0.06 ± 0.01b
	40	0.742 ± 0.014a	0.23 ± 0.02a	0.05 ± 0.02a	0.604 ± 0.016b	0.18 ± 0.02a	0.03 ± 0.01a
	60	0.725 ± 0.009a	0.21 ± 0.01a	0.04 ± 0.01a	0.444 ± 0.018a	0.17 ± 0.01a	0.03 ± 0.01a
R.+LTS	0	**1.544 ± 0.007d**	**0.28 ± 0.02a**	0.07 ± 0.01a	**1.544 ± 0.007d**	**0.28 ± 0.02b**	0.07 ± 0.01a
	20	**1.485 ± 0.023c**	**0.33 ± 0.02b**	0.08 ± 0.02a	**1.244 ± 0.043c**	**0.28 ± 0.01b**	0.08 ± 0.02a
	40	**0.975 ± 0.045b**	**0.33 ± 0.02b**	0.06 ± 0.01a	**0.898 ± 0.024b**	0.22 ± 0.02a	**0.07 ± 0.01a**
	60	**0.782 ± 0.016a**	**0.26 ± 0.02a**	0.06 ± 0.01a	**0.748 ± 0.016a**	**0.20 ± 0.01a**	**0.06 ± 0.01a**
***Hypnum plumaeforme***							
−LTS	0	0.712 ± 0.011c	0.35 ± 0.02b	0.22 ± 0.01b	0.712 ± 0.011d	0.35 ± 0.02c	0.22 ± 0.01c
	20	0.735 ± 0.006c	0.33 ± 0.03ab	0.23 ± 0.01b	0.650 ± 0.013c	0.37 ± 0.02c	0.20 ± 0.02b
	40	0.651 ± 0.016b	0.33 ± 0.02ab	0.19 ± 0.01a	0.473 ± 0.039b	0.31 ± 0.01b	0.19 ± 0.01b
	60	0.545 ± 0.008a	0.32 ± 0.01a	0.20 ± 0.01a	0.258 ± 0.013a	0.27 ± 0.02a	0.16 ± 0.01a
+LTS	0	**0.487 ± 0.014d**	**0.25 ± 0.02b**	**0.29 ± 0.01b**	**0.487 ± 0.014d**	**0.25 ± 0.02c**	**0.29 ± 0.01c**
	20	**0.449 ± 0.014c**	**0.24 ± 0.01b**	**0.26 ± 0.01a**	**0.375 ± 0.038c**	**0.23 ± 0.01c**	**0.27 ± 0.01b**
	40	**0.359 ± 0.024b**	**0.23 ± 0.01b**	**0.25 ± 0.02a**	**0.276 ± 0.018b**	**0.20 ± 0.02b**	0.23 ± 0.03b
	60	**0.293 ± 0.018a**	**0.20 ± 0.01a**	**0.25 ± 0.02a**	**0.205 ± 0.007a**	**0.16 ± 0.01a**	0.15 ± 0.02a
R.−LTS	0	0.713 ± 0.011b	0.34 ± 0.01c	0.22 ± 0.02a	0.713 ± 0.011d	0.34 ± 0.01c	0.22 ± 0.02b
	20	0.715 ± 0.008b	0.35 ± 0.01c	0.20 ± 0.02a	0.658 ± 0.018c	0.36 ± 0.01c	0.20 ± 0.01b
	40	0.676 ± 0.018a	0.30 ± 0.02b	0.21 ± 0.01a	0.588 ± 0.013b	0.32 ± 0.02b	0.16 ± 0.01a
	60	0.663 ± 0.021a	0.27 ± 0.01a	0.18 ± 0.02a	0.520 ± 0.017a	0.23 ± 0.02a	0.14 ± 0.01a
R.+LTS	0	**1.595 ± 0.019d**	**0.55 ± 0.01b**	0.25 ± 0.01a	**1.595 ± 0.019d**	**0.55 ± 0.01b**	0.25 ± 0.01c
	20	**1.431 ± 0.030c**	**0.53 ± 0.03b**	**0.26 ± 0.01a**	**1.380 ± 0.033c**	**0.64 ± 0.03c**	**0.24 ± 0.01bc**
	40	**1.188 ± 0.013b**	**0.56 ± 0.03b**	0.23 ± 0.02a	**1.065 ± 0.034b**	**0.57 ± 0.02b**	**0.22 ± 0.01b**
	60	**1.009 ± 0.011a**	**0.46 ± 0.01a**	**0.22 ± 0.01a**	**0.906 ± 0.013a**	**0.47 ± 0.03a**	**0.20 ± 0.01a**

*Data are presented as means ± S.D. (n = 3). Different letters indicate significant differences between samples exposed to various levels of N addition under each temperature treatment (p < 0.05, one-way ANOVA, LSD test). Data from +LTS and R.+LTS groups in bold format represent significant differences between corresponding +LTS and −LTS, and R.+LTS and R.−LTS samples, respectively (p < 0.05, t-test)*.

After a 10-day recovery period, PIabs in −LTS *P. cirratum* and high N treated −LTS *H. plumaeforme* generally increased significantly. Substantial increases were observed in PIabs and NCPSP in +LTS samples, with greater increases in *H. plumaeforme* (up to 324% for PIabs and 194% for NCPSP) than in *P. cirratum* (up to 159% for PIabs and 136% for NCPSP), with significantly higher values of these indices than those of corresponding R.−LTS samples. Nevertheless, the inhibition effect of N application on PIabs remained apparent and N application sharply decreased its recovery rate. After recovery, the CPSP activity in −N/+LTS *H. plumaeforme* decreased; however, ammonium application slowed the downwards trend (Tables 1, 2).

**Table 2:** Recovery rate of the performance index (PIabs) of photosystem II, and non-cyclic and cyclic photophosphorylation activities (NCPSP and CPSP, respectively) in *Pogonatum cirratum* subsp. *fuscatum* and *Hypnum plumaeforme* exposed to the indicated amount of N addition, with or without low temperature stress after a 10-day-recovery period (+LTS and −LTS respectively).

**N treatment (kg N hm^−2^ year^−1^)**		**Nitrate treatment**			**Ammonium treatment**		
		**PIabs**	**NCPSP (μmol ATP mg^−1^chl min^−1^)**	**CPSP (μmol ATP mg^−1^chl min^−1^)**	**PIabs**	**NCPSP (μmol ATP mg^−1^chl min^−1^)**	**CPSP (μmol ATP mg^−1^chl min^−1^)**
***Pogonatum cirratum* subsp. *fuscatum***							
−LTS	0	**0.021 ± 0.008a**	−0.032 ± 0.030	0.008 ± 0.008	**0.021 ± 0.008a**	−0.032 ± 0.030	0.008 ± 0.008
	20	**0.032 ± 0.005a**	−0.042 ± 0.027	−0.001 ± 0.022	0.061 ± 0.034ab	−0.014 ± 0.021	−0.006 ± 0.015
	40	**0.134 ± 0.004b**	−0.022 ± 0.020	−0.005 ± 0.029	**0.102 ± 0.031bc**	−0.037 ± 0.020	−0.014 ± 0.022
	60	**0.219 ± 0.030c**	−0.027 ± 0.012	−0.007 ± 0.023	**0.115 ± 0.016c**	0.011 ± 0.004	−0.014 ± 0.012
+LTS	0	**0.948 ± 0.024c**	**0.056 ± 0.002a**	−0.024 ± 0.017	**0.948 ± 0.027d**	**0.056 ± 0.002**	−0.024 ± 0.017
	20	**0.905 ± 0.013c**	**0.155 ± 0.021bc**	0.001 ± 0.009	**0.751 ± 0.028c**	**0.083 ± 0.031**	−0.011 ± 0.029
	40	**0.554 ± 0.046b**	**0.184 ± 0.011c**	−0.023 ± 0.011	**0.513 ± 0.019b**	**0.062 ± 0.013**	0.005 ± 0.022
	60	**0.423 ± 0.028a**	**0.121 ± 0.030b**	−0.004 ± 0.006	**0.437 ± 0.013a**	**0.082 ± 0.017**	0.006 ± 0.002
***Hypnum plumaeforme***							
−LTS	0	0.001 ± 0.023a	−0.005 ± 0.005bc	−0.005 ± 0.030	0.001 ± 0.023	−0.005 ± 0.005b	−0.005 ± 0.030
	20	−**0.020 ± 0.007a**	0.012 ± 0.023c	−**0.021 ± 0.005**	0.007 ± 0.013	−0.017 ± 0.022ab	0.007 ± 0.018
	40	0.024 ± 0.032a	−0.034 ± 0.021ab	0.023 ± 0.026	**0.115 ± 0.026**	0.009 ± 0.021b	−**0.024 ± 0.004**
	60	**0.117 ± 0.030b**	−**0.057 ± 0.016a**	−**0.026 ± 0.010**	**0.262 ± 0.004**	−**0.038 ± 0.008a**	−0.008 ± 0.026
+LTS	0	**1.107 ± 0.010d**	**0.293 ± 0.012ab**	−**0.042 ± 0.011**	**1.107 ± 0.010**	**0.293 ± 0.012a**	−**0.042 ± 0.011a**
	20	**0.982 ± 0.033c**	**0.291 ± 0.031a**	0.003 ± 0.028	**1.005 ± 0.013**	**0.409 ± 0.034c**	−**0.029 ± 0.008ab**
	40	**0.829 ± 0.034b**	**0.328 ± 0.014b**	−**0.026 ± 0.004**	**0.789 ± 0.051**	**0.361 ± 0.013b**	−0.009 ± 0.020b
	60	**0.716 ± 0.008a**	**0.264 ± 0.014a**	−**0.022 ± 0.022**	**0.702 ± 0.020**	**0.305 ± 0.030a**	**0.049 ± 0.014c**

*Data are presented as means ± S.D. (n = 3). Different letters indicate significant differences between samples exposed to various levels of N addition under each temperature treatment (p < 0.05, one-way ANOVA, LSD test). Data in bold format represent significant differences with 0 (p < 0.05, t-test)*.

### C assimilation and transformation

#### Effects of N application and LTS

**Figure 1:**
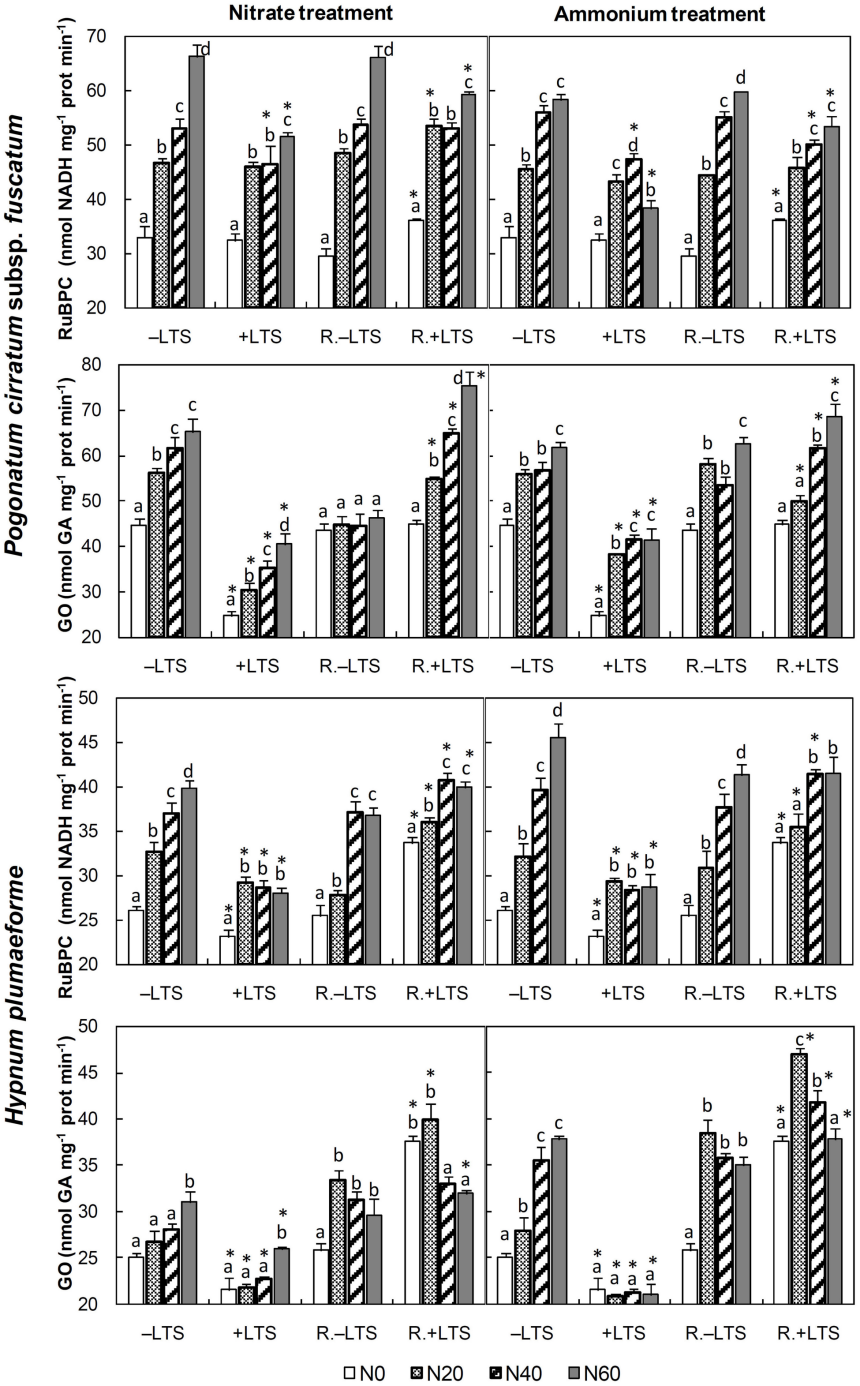
Activities of ribulose-1,5-bisphosphate carboxylase (RuBPC) and glycolate oxidase (GO) in *Pogonatum cirratum* subsp. *fuscatum* and *Hypnum plumaeforme* exposed to the indicated amounts of N addition, with or without low temperature stress immediately (+LTS and −LTS, respectively), or after a 10-day recovery period (R.+LTS and R.−LTS, respectively). Data are presented as means + S.D. *(n* = 3). Different letters on the bars indicate significant differences between samples exposed to various N concentrations under each temperature treatment (*p* < 0.05, one-way ANOVA, LSD test). ^*^ on the bars in +LTS and R.+LTS groups indicate significant differences between corresponding +LTS and −LTS, and R.+LTS and R.−LTS data, respectively (*p* < 0.05, *t*-test).

N application stimulated the activity of RuBPC in both −LTS and +LTS samples. Compared with corresponding −LTS samples, the activity in +LTS *H. plumaeforme* was significantly lower, while that in +LTS *P. cirratum* was reduced only at mid- to high- N conditions (Figure 1).

N application stimulated GO activity in −LTS samples and +LTS *P. cirratum*, while in +LTS *H. plumaeforme*, GO was not as sensitive to N. In addition, LTS substantially inhibited the activity of GO in both species (up to 44%, Figure 1).

**Figure 2:**
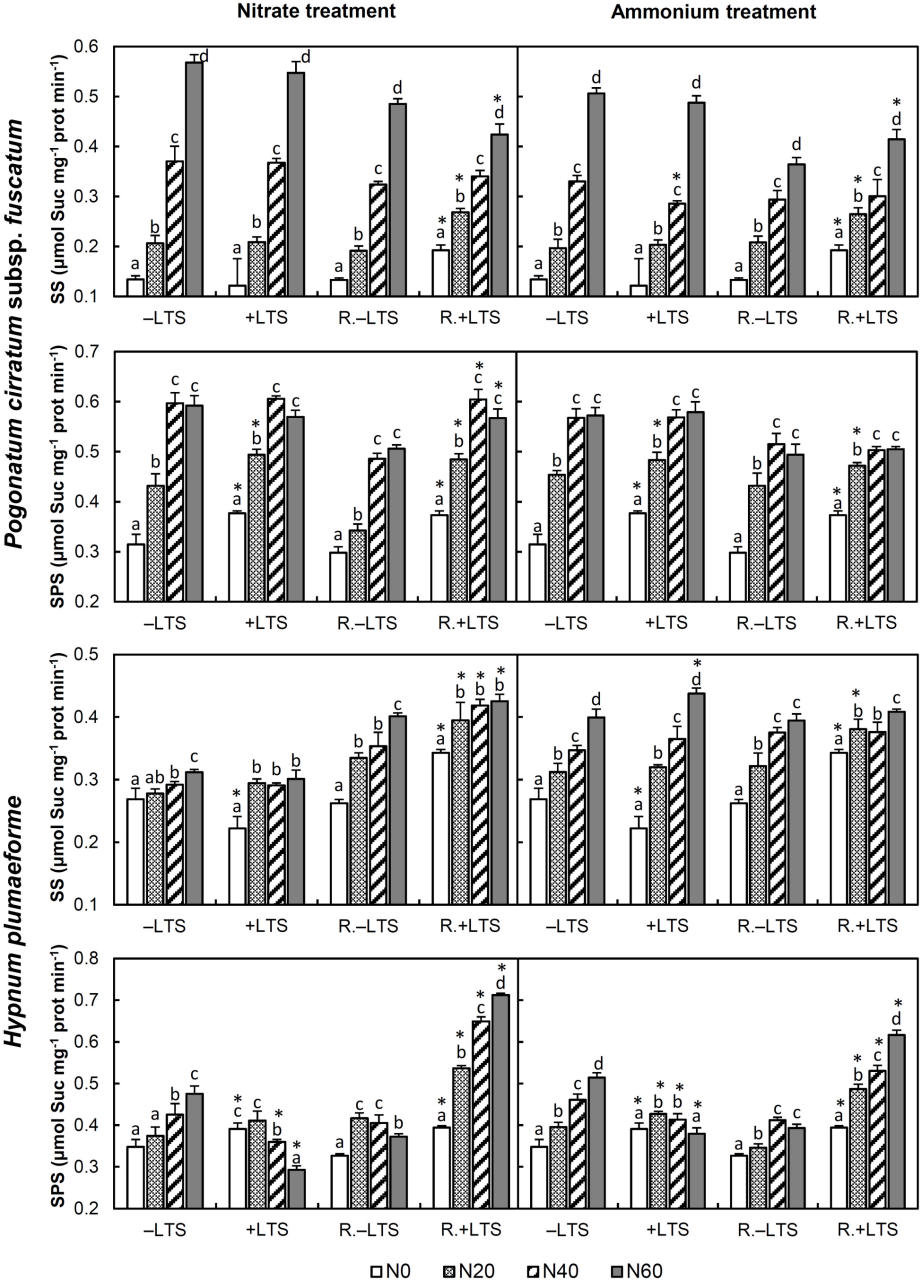
Activities of sucrose-phosphate synthase (SPS) and sucrose synthase (SS) in *Pogonatum cirratum* subsp. *fuscatum* and *Hypnum plumaeforme* exposed to the indicated amounts of N addition, with or without low temperature stress immediately (+LTS and −LTS respectively), or after a 10-day recovery period (R.+LTS and R.−LTS, respectively). Data are presented as means + S.D. (*n* = 3). Different letters on the bars indicate significant differences between samples exposed to various N treatment concentrations under each temperature treatment (*p* < 0.05, one-way ANOVA, LSD test). ^*^ on the bars in +LTS and R.+LTS groups indicate significant differences between corresponding +LTS and −LTS, R.+LTS and R.−LTS data, respectively (*p* < 0.05, *t*-test).

N application stimulated the activities of SS and SPS in −LTS samples and +LTS *P. cirratum*. In +LTS *H. plumaeforme*, SS was also stimulated by N addition; however, SPS was only stimulated by low- to mid-ammonium addition and was inhibited by mid- to high-nitrate N addition. The differences between SS in +LTS and −LTS samples were not particularly pronounced, while SPS activity was higher in +LTS samples than that in corresponding −LTS samples at −N or low N application conditions, and similar to or lower than corresponding −LTS samples exposed to higher N addition (Figure 2).

#### Changes after recovery

After a short-term recovery, RuBPC activities in +LTS *H. plumaeforme* recovered significantly, but its recoveries in +LTS *P. cirratum* were not always significant. RuBPC activities were higher in R.+LTS than corresponding R.−LTS samples without additional N; however, N application reduced the difference, and RuBPC activity in R.+LTS *P. cirratum* was even lower than that in corresponding R.−LTS samples under high nitrate, or mid-to-high ammonium conditions. GO activity in +LTS samples exhibited a sharp rise after recovery and was generally higher than that in corresponding R.−LTS samples (Figure 1, Table 3).

**Table 3:** Recovery rate of ribulose-1,5-bisphosphate carboxylase (RuBPC) and glycolate oxidase (GO) in *Pogonatum cirratum* subsp. *fuscatum* and *Hypnum plumaeforme* exposed to the indicated amount of N addition, with or without low temperature stress after a 10-day-recovery period (+LTS and −LTS respectively).

**N treatment (kg N ha^−1^)**		**Nitrate teatment**		**Ammonium treatment**	
		**RuBPC (nmol CO_2_ mg^−1^prot min^−1^)**	**GO (nmol GA mg^−1^prot min^−1^)**	**RuBPC (nmol CO_2_ mg^−1^prot min^−1^)**	**GO (nmol GA mg^−1^prot min^−1^)**
***Pogonatum cirratum* subsp. *fuscatum***					
−LTS	0	−**3.449 ± 0.554a**	−1.047 ± 4.151b	−**3.449 ± 0.554a**	−1.047 ± 4.151
	20	1.797 ± 1.707b	−11.562 ± 4.983a	−1.148 ± 1.127ab	2.108 ± 5.504
	40	0.849 ± 1.085b	−**16.975 ± 5.067a**	−0.775 ± 2.257ab	−3.082 ± 3.026
	60	−0.071 ± 4.246b	−**18.840 ± 1.755a**	1.475 ± 1.299b	0.759 ± 1.654
+LTS	0	3.723 ± 1.574	**20.017 ± 1.828a**	3.723 ± 1.574a	**20.017 ± 1.828b**
	20	**7.284 ± 2.009**	**24.414 ± 1.075ab**	2.428 ± 2.127a	**11.732 ± 1.768a**
	40	6.700 ± 2.728	**29.331 ± 1.948bc**	2.672 ± 1.710a	**20.298 ± 3.126b**
	60	**7.737 ± 0.924**	**34.836 ± 6.797c**	**15.142 ± 0.559b**	**27.155 ± 6.423b**
***Hypnum plumaeforme***					
−LTS	0	−0.655 ± 1.647b	0.750 ± 0.922ab	−0.655 ± 1.647	0.750 ± 0.922a
	20	−**4.876 ± 0.763a**	**6.665 ± 0.387c**	−1.304 ± 2.642	**10.531 ± 2.721b**
	40	0.246 ± 0.529b	3.189 ± 1.657b	−1.886 ± 1.512	0.238 ± 2.141a
	60	−3.085 ± 1.250a	−1.385 ± 2.975a	−4.237 ± 2.623	−2.838 ± 1.607a
+LTS	0	**10.524 ± 1.347b**	**16.013 ± 1.390b**	**10.524 ± 1.347a**	**16.013 ± 1.390a**
	20	**6.844 ± 1.225a**	**18.202 ± 3.616b**	**6.177 ± 1.098b**	**26.165 ± 1.408b**
	40	**11.997 ± 0.814b**	**10.256 ± 2.061a**	**13.135 ± 1.118 b**	**20.570 ± 3.377a**
	60	**11.887 ± 0.748b**	**5.973 ± 1.543a**	**12.840 ± 2.526b**	**16.798 ± 3.253a**

*Data are presented as means ± S.D. (n = 3). Different letters indicate significant differences between samples exposed to various levels of N addition under each temperature treatment (p < 0.05, one-way ANOVA, LSD test). Data in bold format represent significant differences with 0 (p < 0.05, t-test)*.

After recovery, SPS activity in mid-to-high N treated −LTS *P. cirratum* and hight N treated −LTS *H. plumaeforme* decreased significantly. SS activity in +LTS *P. cirratum* increased at −N or low N application conditions and was higher than that of corresponding R.−LTS samples; however, SS activity did not change significantly, or even decreased, in samples treated with mid- to high-N levels. SS activity in +LTS *H. plumaeforme* also increased after recovery and was higher than that in corresponding R.−LTS samples, except under conditions of mid and high ammonium application. Although SPS activity in +LTS *P. cirratum* did not rise after recovery, it was higher than that in corresponding R.−LTS samples, except under conditions of mid- to high-ammonium application. Meanwhile, SPS activity in +N/+LTS *H. plumaeforme* increased after recovery, with the recovery rate elevating sharply with increased levels of N addition rate (up to 143 and 63% for high nitrate and high ammonium treated samples, respectively), and was significantly higher than that of corresponding R.−LTS samples (Figure 2, Table S1).

### N assimilation and transformation

#### Effects of N application and LTS

**Figure 3:**
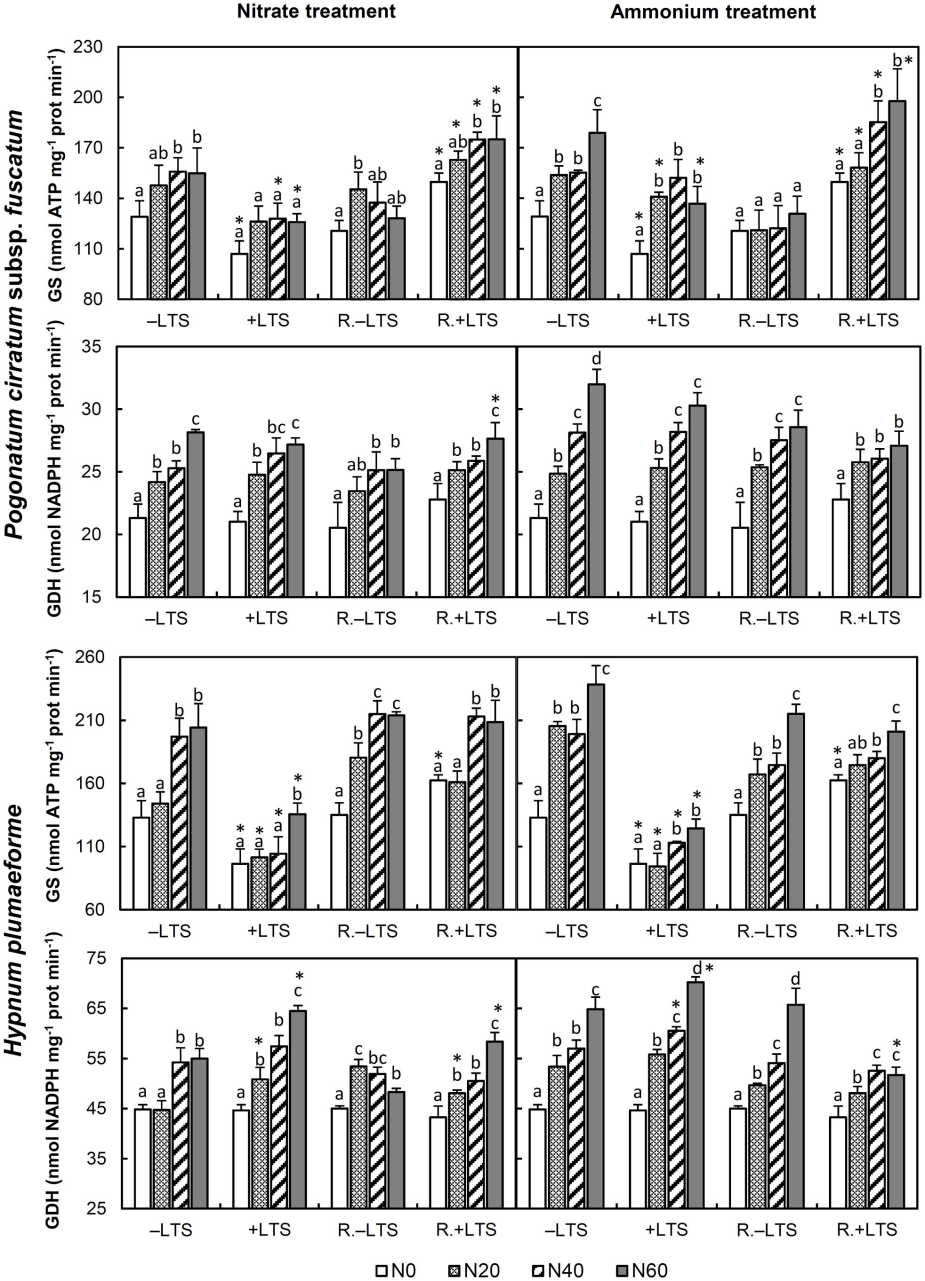
Activities of glutamine synthetase (GS) and glutamate dehydrogenase (GDH) in *Pogonatum cirratum* subsp. *fuscatum* and *Hypnum plumaeforme* exposed to the indicated amounts of N addition, with or without low temperature stress immediately (+LTS and −LTS respectively), or after a 10-day recovery period (R.+LTS and R.−LTS, respectively). Data are presented as means + S.D. (*n* = 3). Different letters on the bars indicate significant differences between samples exposed to various N concentrations under each temperature treatment (*p* < 0.05, one-way ANOVA, LSD test). ^*^ on the bars in the +LTS and R.+LTS groups indicate significant differences between corresponding +LTS and −LTS, and R.+LTS and R.−LTS data, respectively (*p* < 0.05, *t*-test).

**Figure 4:**
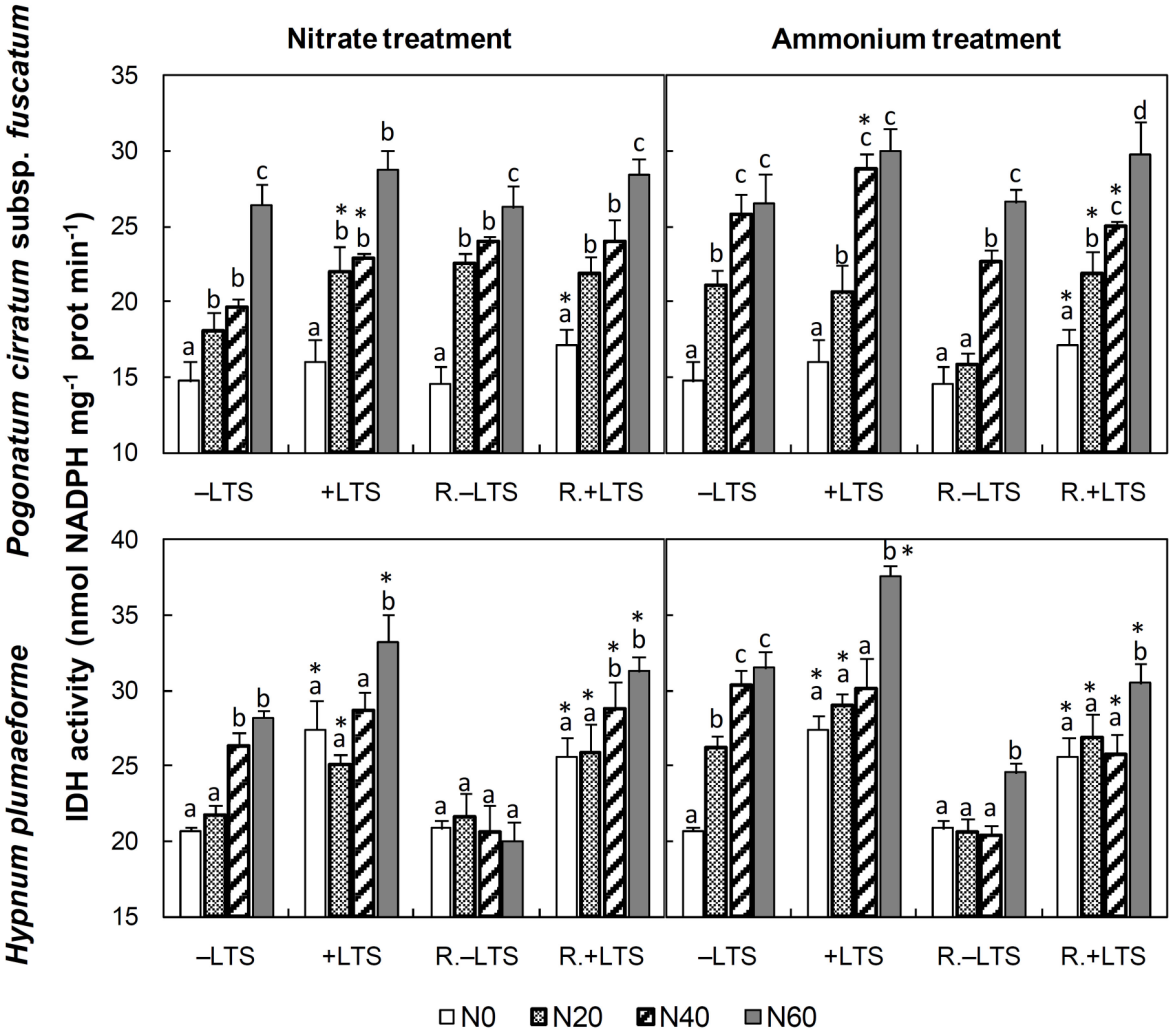
Activities of NADP-isocitrate dehydrogenase (IDH) in *Pogonatum cirratum* subsp. *fuscatum* and *Hypnum plumaeforme* exposed to the indicated amounts of N addition, with or without low temperature stress immediately (+LTS and −LTS respectively), or after a 10-day recovery period (R.+LTS and R.−LTS, respectively). Data are presented as means + S.D. (*n* = 3). Different letters on the bars indicate significant differences between samples exposed to various N concentrations under each temperature treatment (*p* < 0.05, one-way ANOVA, LSD test). ^*^ on the bars in the +LTS and R.+LTS groups indicate significant differences between the corresponding +LTS and −LTS, and R.+LTS and R.−LTS data respectively (*p* < 0.05, *t*-test).

N addition stimulated the activities of GS, GDH and IDH in −LTS samples (Figures 3, 4). LTS led to decreased GS activity, and decreased sensitivity of GS to N addition, particularly in *H. plumaeforme*. However, the effect of LTS on GDH in *P. cirratum* was not significant and, under +N conditions, it even stimulated GDH activity in *H. plumaeforme* (Figure 3). In general, LTS also stimulated IDH activity in *H. plumaeforme*; however, in *P. cirratum* IDH was only stimulated by LTS under low N and medium nitrate application conditions (Figure 4).

#### Changes after recovery

After recovery, GS in ammonium treated *P. cirratum* and low-to-mid nitrate and low ammonium treated −LTS *H. plumaeforme* decreased significantly. GS activity, which was inhibited in +LTS samples, rose significantly after recovery generally, and GS activity in R.+LTS *P. cirratum* was significantly higher than that in corresponding R.−LTS samples. However, GS activity in R.+LTS *H. plumaeforme* was higher than that in corresponding R.−LTS samples only in the −N group (Figure 3). After recovery, the activity of GDH in +LTS *P. cirratum* did not change significantly except under mid nitrate and high ammonium addition conditions, while in ammonium treated +LTS *H. plumaeforme*, where GDH activity was stimulated, it decreased after recovery (Figure 3, Table S2).

After recovery, the activity of IDH in +LTS *P. cirratum* generally did not exhibit significant change; however, it was higher than that in corresponding R.−LTS samples under −N and low- to mid-ammonium application conditions. IDH activity in +LTS *H. plumaeforme* decreased under conditions of mid- and high-ammonium addition, and it was also significantly higher than that in corresponding R.−LTS samples, and the stimulating effect of N addition remained. Of particular note, the stimulatory effect of N addition on IDH in −LTS *H. plumaeforme* almost completely disappeared after the recovery period (Figure 4, Table S2).

### N and FAA content

#### Effects of N application and LTS

**Figure 5:**
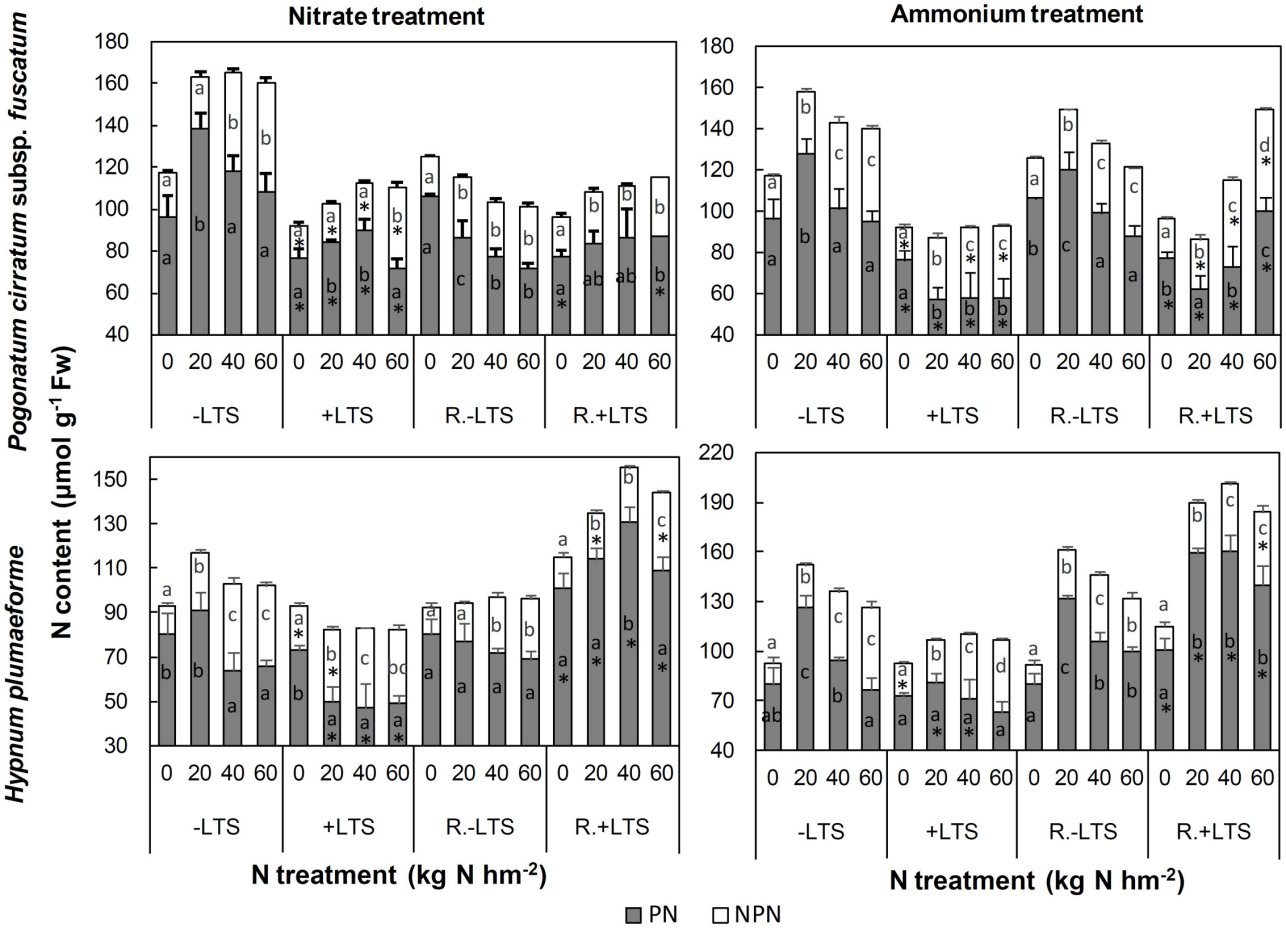
Protein-N (PN) and non-protein-N (NPN) contents in *Pogonatum cirratum* subsp. *fuscatum* and *Hypnum plumaeforme* exposed to the indicated amounts of N addition, with or without low temperature stress immediately (+LTS and −LTS, respectively), or after a 10-day recovery period (R.+LTS and R.−LTS, respectively). Data are presented as means ± S.D. (*n* = 3). Different letters on the bars indicate significant differences between samples exposed to various N treatment concentrations under each temperature treatment (*p* < 0.05, one-way ANOVA, LSD test). ^*^on the bars in the +LTS and R.+LTS groups represent significant differences between corresponding +LTS and −LTS, and R.+LTS and R.−LTS data respectively (*p* < 0.05, *t*-test).

N application significantly stimulated the accumulation of NPN in both −LTS and +LTS samples. LTS led to decreases in PN and NPN content in *P. cirratum*; however, in *H. plumaeforme*, LTS stimulated the accumulation of NPN under −N and low nitrate conditions and inhibited the accumulation of PN under +N conditions (Figure 5).

**Figure 6:**
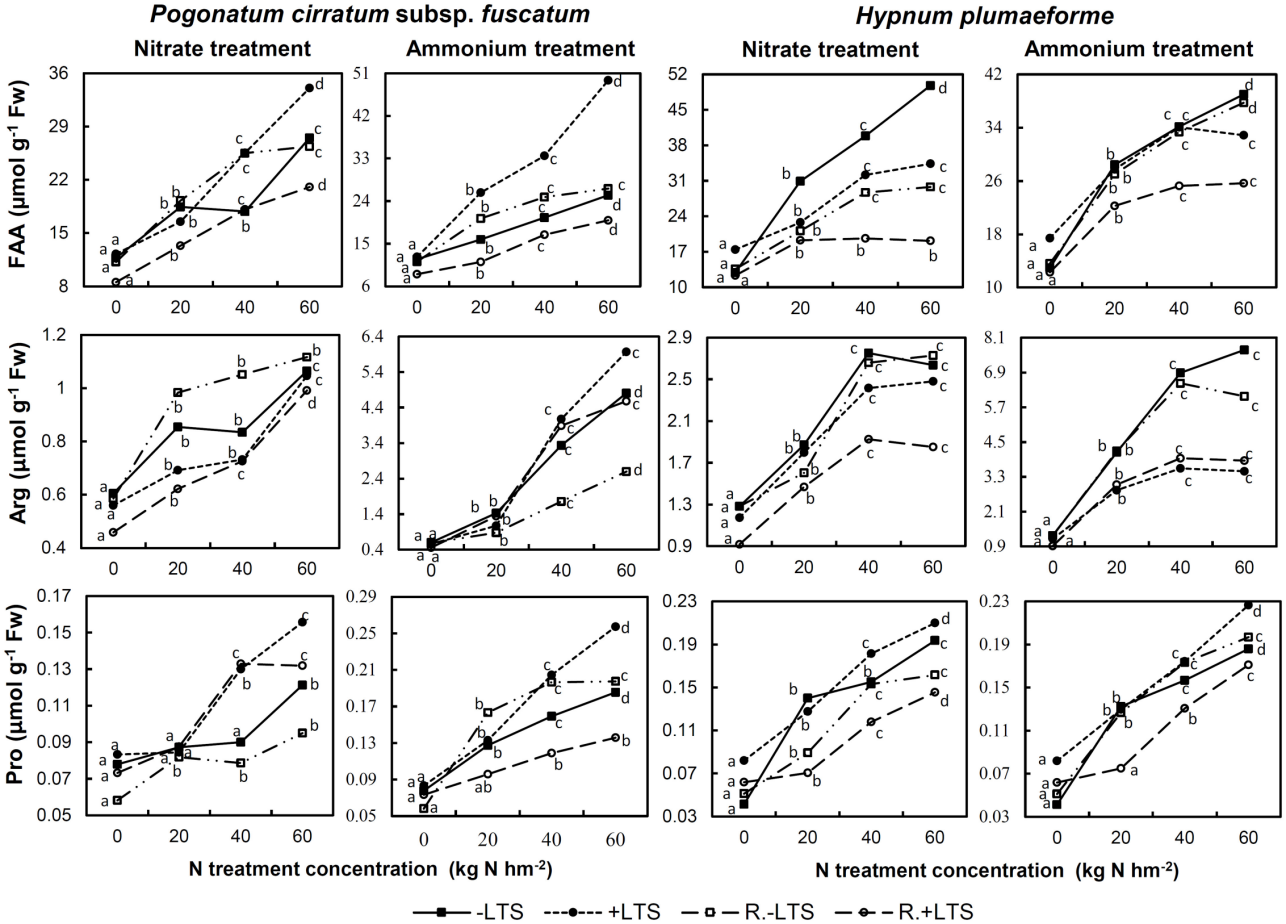
Total free amino acids (FAA), arginine (Arg), and proline (Pro) contents in *Pogonatum cirratum* subsp. *fuscatum* and *Hypnum plumaeforme* exposed to the indicated amounts of N addition, with or without low temperature stress immediately (+LTS and −LTS, respectively), or after a 10-day recovery period (R.+LTS and R.−LTS, respectively). For each treatment, different letters beside the lines indicate significant differences between samples exposed to various N concentrations under each temperature treatment (*p* < 0.05, one-way ANOVA, LSD test). For clarity, standard deviations are not presented.

N addition also stimulated the accumulation of total FAA, Arg, and Pro in both −LTS and +LTS samples. The effects of LTS on FAA in −N samples were not significant; however, combined N application and LTS led to increased Pro content in both species, while its effects on total FAA and Arg varied with species and N treatment (Figure 6).

#### Changes after recovery

After recovery, NPN in mid-to-high nitrate and high ammonium treated −LTS samples decreased significantly. The change of PN content in +LTS *P. cirratum* was not significant after recovery, other than an increase of 72% in samples treated with high ammonium. In +LTS *H. plumaeforme* PN increased substantially (up to 179 and 125% for nitrate and ammonium treated samples, respectively), and was higher than in corresponding R.−LTS samples (Figure 5, Table S3).

In +LTS samples, total FAA generally exhibited a decreasing trend after recovery, although changes of Arg and Pro in +LTS samples varied with species and N treatment. In addition, total FAA, Arg, and Pro contents of R.+LTS *H. plumaeforme* were lower than those in corresponding R.−LTS samples, while for *P. cirratum*, only total FAA content exhibited a similar trend (Figure 6, Table S4).

## Discussion

### PSII performance and photophosphorylation

PIabs is a comprehensive index characterizing the function of PSII. In the present study, N application decreased PIabs, NCPSP, and CPSP significantly, which might be due to the oxidative stress (Liu et al., 2015) and increased chlorophyll contents (Figure S2) caused by N addition. In addition, the decrease of RC in unit area (data not shown) might also contribute to the sharp decrease of PIabs. However, ammonium caused stronger declines in PIabs than nitrate and similar results were found for NCPSP and CPSP, which was in accordance with our previous study (Liu et al., 2016). LTS caused significant decreases in PIabs in mosses, and combined LTS and N treatment induced a further decrease, due to the oxidative stress caused by LTS and excess N (Liu et al., 2015), resulting in damage to PSII (Allen and Ort, 2001; Duan et al., 2012). Moreover, LTS stimulated CPSP activity, but inhibited NCPSP activity, in the mosses. As cyclic electron transfer is a candidate for chill-induced electron sink and can prevent over reduction of the P700 reaction centers (Allen and Ort, 2001; Kono et al., 2014), the shift from NCPSP to CPSP is likely to be an important photoprotection strategy for mosses exposed to LTS.

Overcompensation of PSII function was observed in both species. Compensation effects of PSII function have also been reported in seed plants, although recovery ability was closely related to species and the intensity of stress (Gesch and Heilman, 1999; Ploschuk et al., 2014). The stress temperature used in the present study was higher than the lowest temperature in the studied area; hence the damage caused by LTS in the mosses was reversible, permitting PSII function overcompensation. As expected, nitrogen addition decreased the compensation ability of PSII function in the mosses.

### Photosynthetic carbon assimilation and carbohydrate synthesis

Our results showed that N application stimulated the activity of RuBPC, probably due to the enhanced synthesizing of Rubisco at N addition conditions (Guo et al., 2005). As RuBPC is the rate limiting reaction in C assimilation, the increased RuBPC activity at N addition conditions might suggest an increased C assimilation in N treated mosses. However, the following reactions of C assimilation that need ATP as energy and substrate might be affected by the decreased PIabs, NCPSP and CPSP at N addition conditions, though the decrease of ATP synthesize by NCPSP and CPSP might not be so pronounce due to the increase of chlorophyll. As a result, net photosynthesis rate was generally mildly increased by N application (Liu et al., 2011).

The decreased RuBPC activity in mosses under +LTS conditions is an indication that C assimilation was inhibited, consistent with the reports in seed plants (Hutchison et al., 2000; Zhang et al., 2002; Abat and Deswal, 2009). Photorespiration is an important defense mechanism against photo damage in plants, with GO a key enzyme in the process (Wingler et al., 2000). Mosses can increase photorespiration to mitigate photo damage under high nitrogen and drought stress conditions (Liu et al., 2016). The GO activity increased with the increase in N addition rate, which was consistent with expectations. However, we found that LTS inhibited GO activity in mosses, indicating that mechanisms other than photorespiration are employed by these plants to combat photo damage at low temperatures (Allen and Ort, 2001), which differs from their responses to drought stress.

SPS is a key enzyme in the sucrose biosynthesis pathway and SS participates in the degradation of sucrose (Touchette and Burkholder, 2007). N application stimulated the activities of SS and SPS in the present study, which indicated that N stimulated the transfer and utilization of sucrose. LTS mildly stimulated the activity of SPS in −N samples; however, a combination of mid- to high-N treatment and LTS caused a decrease in SPS, similar to responses under drought conditions (Liu et al., 2016). Unlike drought stress, LTS did not inhibit SS activity. In addition, LTS generally caused a decline in starch content and an increase in TSS in the mosses, particularly under +N conditions (Table S5), suggesting that the mosses consumed starch as a C skeleton to maintain both N assimilation and a relatively high TSS concentration under +LTS conditions.

After recovery, RuBPC activity in the +LTS mosses increased sharply, suggesting compensatory photosynthetic C assimilation in both species, which declined in intensity with increasing levels of N addition. Compensation effects were also found for SPS and SS activity. The compensation intensity of SS and SPS in *H. plumaeforme* was generally higher than that in *P. cirratum* under +N conditions, suggesting that the recovery of sucrose transfer and utilization in *H. plumaeforme* was superior to that of *P. cirratum*, which may be related to the higher intensity of protein synthesis compensation in this species (see below). In addition, although GO activity was substantially inhibited by LTS, its activity increased sharply after recovery, indicating the importance of GO in damage repair during the process of recovery after LTS.

### N metabolism

Both the glutamine synthetase/glutamate synthase (GS/GOGAT) and GDH pathways are primary ammonium assimilation pathways in plants. The reaction catalyzed by IDH is not only the rate-limiting step of the tricarboxylic acid cycle, it also provides carbon skeletons for amino acid synthesis, and contributes to plant defense mechanisms through recycling NADPH (Lu et al., 2005; Valderrama et al., 2006). In respond to N additions, GS, GDH, and IDH activities in the mosses increased in the present manuscript. LTS causes increases in both GS content and activity, while it led to a decline in GDH activity in rice (Lu et al., 2005; Yan et al., 2006). In contrast, our results indicate that the GS/GOGAT pathway was inhibited by LTS in the mosses (see Figure S3 for GOGAT data), while the GDH pathway was maintained at stable or slightly increased under +LTS conditions, exhibiting a significantly different response of the ammonium assimilation pathway to LTS compared with rice, which also differed in their response to drought (Liu et al., 2016). Thus, responses of the two inorganic N assimilation pathways to different stressors are related to both species and types of stress. The maintenance of relatively high GDH activity under +LTS conditions in both moss species suggests that they can guarantee the assimilation of Glu, which is the precursor for synthesis of numerous substances involved in cold-resistance, including Arg, Pro, γ-aminobutyric acid, and polyamine (Yoshiba et al., 1997; Paschalidis and Roubelakis-Angelakis, 2005; Mazzucotelli et al., 2006; Alcázar et al., 2011). IDH activity in the mosses sustained relatively high levels or even increased under +LTS conditions, favoring initiation of defensive reactions in the mosses. Furthermore, Pro is an indicator of osmotic stress and LTS only significantly stimulated the accumulation of Pro when applied in combination with N treatment, indicating that N treatment decreased the resistance of the mosses to LTS.

PN content also declined in the mosses under +LTS conditions, indicating that LTS can enhance proteolysis, which is associated with oxidative stress (Palma et al., 2002; Yan et al., 2006).

Compensation effects were also observed in ammonium assimilation in the mosses. PN content increased substantially after recovery in +LTS *H. plumaeforme*, while the change in PN content in +LTS *P. cirratum* was much less pronounced, indicating a higher compensation effect on protein synthesis in *H. plumaeforme* than *P. cirratum*, in accordance with the results of carbon assimilation (see above).

After recovery, the total FAA content of +LTS samples declined, possibly due to reduced stress intensity and enhanced protein synthesis during recovery (Good and Zaplachinski, 1994; Liu et al., 2015). However, IDH activity remained relatively high, partly because of the increase in metabolism and energy requirements during recovery. Moreover, the observed relatively high levels of IDH activity may benefit the mosses by preventing secondary oxidative stress during recovery. Another interesting finding was the sharp decrease of IDH activity in nitrate treated −LTS *H. plumaeforme*, which suggested that the stimulation of IDH by nitrate in −LTS *H. plumaeforme* may be short-lived.

## Conclusion

LTS damaged PSII and inhibited the activities of NCPSP and RuBPC in *P. cirratum* and *H. plumaeforme*, ultimately leading to a substantial decrease in the C assimilation rate. In addition, combined LTS and N application exacerbated the damage to PSII and the inhibition of C assimilation. On the other hand, the mosses used several mechanisms to cope with LTS; for example, the activities of CPSP and IDH were increased to ensure the supply of ATP and NADPH, the turnover rate of sugars was accelerated to ensure C skeleton availability, GDH activity was sustained or increased to ensure Glu supply, and Pro was accumulated to cope with osmotic stress generated by LTS. However, LTS caused a sharp decrease in GO, which is contrary to the responses of mosses to drought stress, suggesting differences in the stress resistance mechanisms to drought and LTS. Divergent responses of the mosses to LTS and drought were also found with respect to the activities of the two ammonium assimilation pathways, GS/GOGAT and GDH.

Both mosses exhibited compensation effects in C and N assimilation after a short-term recovery; however, the extent of these effects decreased in response to combined LTS and N application. In contrast, the inhibited GO activity was substantially restored after recovery, indicating that it has important roles in antioxidation during the recovery period. Furthermore, *H. plumaeforme* exhibited stronger compensation effects in PSII performance, photophosphorylation, C assimilation, and PN accumulation than *P. cirratum*, particularly under +N conditions, indicating that *H. plumaeforme* is more resilient to temperature and N variation than *P. cirratum*, consistent with its wider distribution.

## Author contributions

BL carried out the study, including laboratory experiments, data analysis, and drafting the manuscript. WL was responsible for the design and implementation of the entire study. CL collected the weather data during the study period and participated in the fieldwork and laboratory experiments.

### Conflict of interest statement

The authors declare that the research was conducted in the absence of any commercial or financial relationships that could be construed as a potential conflict of interest.

## Abbreviations

LTS: low temperature stress
−N: without nitrogen addition
+N: with nitrogen addition.
