# Supplementary material for: Nitrogen Addition Exacerbates the Negative Effects of Low Temperature Stress on Carbon and Nitrogen Metabolism in Moss
Source: Front Plant Sci. 2017 Aug 2;8:1328. doi: 10.3389/fpls.2017.01328 (PMC5539086; doi:10.3389/fpls.2017.01328)
Supplement: Supplementary file 1 [file Table1.DOCX]

**Table S1** Recovery rate of sucrose-phosphate synthase (SPS) and sucrose synthase (SS) in *Pogonatum cirratum* subsp. *fuscatum* and *Hypnum plumaeforme* exposed to the indicated amount of N addition, with or without low temperature stress after a 10-day-recovery period (+LTS and –LTS respectively). Data are presented as means ± S.D. (n=3). Different letters indicate significant differences between samples exposed to various levels of N addition under each temperature treatment (*p* < 0.05, one-way ANOVA, LSD test). Data in bold format represent significant differences with 0 (*p* < 0.05, t-test).

| N treatment  (kg N ha^-1^) | | Nitrate teatment | | Ammonium treatment | |
| --- | --- | --- | --- | --- | --- |
|  |  | SS  (μmol Suc mg^-1^ prot min^-1^) | SPS  (μmol Suc mg^-1^ prot min^-1^) | SS  (μmol Suc mg^-1^ prot min^-1^) | SPS  (μmol Suc mg^-1^ prot min^-1^) |
| ***Pogonatum cirratum* subsp. *fuscatum*** | | | | | |
| -LTS | 0 | -0.001±0.007c | -0.017±0.032b | -0.001±0.007c | -0.017±0.032b |
|  | 20 | -0.014±0.020bc | -0.090±0.037a | 0.011±0.025c | -0.022±0.032b |
|  | 40 | -0.046±0.028b | **-0.110±0.024a** | **-0.036±0.013b** | **-0.053±0.015ab** |
|  | 60 | **-0.082±0.012a** | **-0.086±0.020a** | **-0.142±0.003a** | **-0.079±0.029a** |
| +LTS | 0 | **0.071±0.027c** | -0.004±0.012 | **0.071±0.027c** | -0.004±0.012b |
|  | 20 | **0.060±0.009c** | -0.010±0.022 | **0.062±0.010bc** | -0.011±0.011b |
|  | 40 | -0.028±0.020b | -0.002±0.020 | 0.015±0.039b | **-0.065±0.014a** |
|  | 60 | **-0.123±0.024s** | -0.002±0.031 | -0.073±0.031a | **-0.074±0.017a** |
| ***Hypnum plumaeforme*** | | | | | |
| -LTS | 0 | -0.007±0.018a | -0.021±0.020b | -0.007±0.018 | -0.021±0.020b |
|  | 20 | **0.057±0.004b** | 0.043±0.031c | 0.010±0.027 | **-0.049±0.008b** |
|  | 40 | 0.062±0.026bc | -0.020±0.024b | 0.028±0.012 | -0.049±0.021b |
|  | 60 | **0.089±0.010c** | **-0.103±0.016a** | -0.005±0.023 | **-0.121±0.021a** |
| +LTS | 0 | **0.121±0.020** | 0.004±0.016a | **0.121±0.020d** | 0.004±0.016a |
|  | 20 | **0.100±0.035** | **0.125±0.025b** | **0.060±0.018c** | **0.053±0.011b** |
|  | 40 | **0.127±0.011** | **0.289±0.009c** | 0.011±0.020b | **0.117±0.018c** |
|  | 60 | **0.125±0.016** | **0.420±0.006d** | -0.029±0.012a | **0.238±0.021d** |

**Table S2** Recovery rate of glutamine synthetase (GS), glutamate dehydrogenase (GDH) and NADP-isocitrate dehydrogenase (IDH) in *Pogonatum cirratum* subsp. *fuscatum* and *Hypnum plumaeforme* exposed to the indicated amount of N addition, with or without low temperature stress after a 10-day-recovery period (+LTS and –LTS respectively). Data are presented as means ± S.D. (n=3). Different letters indicate significant differences between samples exposed to various levels of N addition under each temperature treatment (*p* < 0.05, one-way ANOVA, LSD test). Data in bold format represent significant differences with 0 (*p* < 0.05, t-test).

| N treatment  (kg N hm^-2^ yr^-1^) | | Nitrate treatment | | | Ammonium treatment | | |
| --- | --- | --- | --- | --- | --- | --- | --- |
|  |  | GS  (nmol ATP mg^-1^ prot min^-1^) | GDH  (nmol NADPH mg^-1^ prot min^-1^) | IDH  (nmol NADPH mg^-1^ prot min^-1^) | GS  (nmol ATP mg^-1^ prot min^-1^) | GDH  (nmol NADPH mg^-1^ prot min^-1^) | IDH  (nmol NADPH mg^-1^ prot min^-1^) |
| ***Pogonatum cirratum* subsp. *fuscatum*** | | | | | | |  |
| -LTS | 0 | -9.229±7.510 | -0.778±0.898 | -0.176±2.371a | -9.229±7.510b | -0.778±0.898b | -0.176±2.371b |
|  | 20 | -2.221±16.826 | -0.715±1.996 | **4.433±0.800b** | **-32.957±10.530a** | 0.513±0.784b | **-5.234±1.654a** |
|  | 40 | -18.361±12.228 | -0.157±2.022 | **4.358±0.379b** | **-33.066±12.593a** | -0.621±1.582b | **-3.052±0.866ab** |
|  | 60 | -26.563±19.113 | **-2.988±1.105** | -0.148±2.411a | **-48.139±9.714a** | -3.412±1.949a | 0.035±2.144b |
| +LTS | 0 | **42.687±7.433** | 1.766±1.913 | 1.105±0.906 | **42.687±7.433ab** | 1.766±1.913b | 1.105±0.906b |
|  | 20 | **36.607±13.439** | 0.347±0.700 | -0.115±2.444 | 17.428±11.299a | 0.459±1.625b | 1.322±0.756b |
|  | 40 | **47.150±10.965** | **-0.581±0.162** | 1.067±1.552 | **33.266±9.673ab** | -2.080±0.819a | **-3.773±0.150a** |
|  | 60 | **49.073±9.376** | 0.482±1.555 | -0.303±1.101 | 60.938±29.279b | **-3.222±0.370a** | -0.237±2.311b |
| ***Hypnum plumaeforme*** | | | | | | |  |
| -LTS | 0 | 2.278±3.936a | 0.216±1.381b | 0.171±0.795b | 2.278±3.936b | 0.216±1.381 | 0.171±0.795c |
|  | 20 | **36.313± 9.337b** | **8.697±1.868c** | -0.162± 1.785b | **-38.215±9.084a** | -3.714±1.885 | **-5.664±1.446b** |
|  | 40 | **17.877±4.248ab** | -2.326±1.794b | **-5.680± 1.922a** | -24.449±19.960a | **-2.924±0.704** | **-9.945±0.672a** |
|  | 60 | 9.543±20.986a | -6.687±3.347a | **-8.163±1.682a** | -23.298±16.683ab | 0.244±6.550 | **-6.934±1.166a** |
| +LTS | 0 | **66.081±21.071a** | -1.376±2.128 | -1.794±3.053 | **66.081±21.071** | -1.376±2.128c | -1.794±3.035b |
|  | 20 | **59.595±6.549a** | -2.756±2.816 | 0.706±2.192 | **80.105±10.887** | **-7.739± 2.267b** | -2.162±2.020b |
|  | 40 | **108.564±3.006b** | -0.944±3.076 | 0.075±1.660 | **67.017±5.133** | **-7.954±1.890b** | **-4.363±1.502ab** |
|  | 60 | **73.244±26.011a** | -6.082±2.919 | -1.945±2.385 | **76.549±12.905** | **-18.534±0.907a** | **-7.085±2.528a** |

**Table S3** Recovery rate of Protein-N (PN) and non-protein-N (NPN) contents in *Pogonatum cirratum* subsp. *fuscatum* and *Hypnum plumaeforme* exposed to the indicated amount of N addition, with or without low temperature stress after a 10-day-recovery period (+LTS and –LTS respectively). Data are presented as means ± S.D. (n=3). Different letters indicate significant differences between samples exposed to various levels of N addition under each temperature treatment (*p* < 0.05, one-way ANOVA, LSD test). Data in bold format represent significant differences with 0 (*p* < 0.05, t-test).

| N treatment  (kg N ha^-1^) | | Nitrate teatment | | Ammonium treatment | |
| --- | --- | --- | --- | --- | --- |
|  |  | NPN  (μmol g^-1^ Fw) | PN  (μmol g^-1^ Fw) | NPN  (μmol g^-1^ Fw) | PN  (μmol g^-1^ Fw) |
| ***Pogonatum cirratum* subsp. *fuscatum*** | | | | | |
| -LTS | 0 | -1.859±3.858b | 10.260±8.774b | -1.859±3.858b | 10.260±8.774 |
|  | 20 | **4.047±1.140b** | **-51.755±8.588a** | -1.151±0.503b | -8.740±15.924 |
|  | 40 | **-20.666±7.629a** | -40.048±16.788a | -7.957±3.211a | -1.087±6.077 |
|  | 60 | **-22.228±5.594a** | -37.642±20.628a | **-12.214±1.976a** | -6.250±9.159 |
| +LTS | 0 | 2.677±1.466b | 0.753±3.254a | 2.677±1.466c | 0.753±3.254 |
|  | 20 | **6.771±1.543b** | -0.592±6.229a | **-15.492±3.349a** | 4.415±11.557 |
|  | 40 | 2.918±4.789b | -4.057±7.741a | -2.083±2.365b | 14.334±20.723 |
|  | 60 | -10.460±11.619a | 15.622±5.947b | 3.576±1.708c | **41.658±7.477** |
| ***Hypnum plumaeforme*** | | | | | |
| -LTS | 0 | -0.803±3.935b | 0.103±3.884 | -1.214±6.116b | 0.103±3.884a |
|  | 20 | -9.144± 9.106ab | -13.407±14.062 | 3.235±2.250b | 6.040±16.350ab |
|  | 40 | **-14.419±4.064a** | 8.520±7.852 | -2.137±4.556b | 12.093±7.982ab |
|  | 60 | **-8.991±2.229ab** | 2.839±5.137 | **-16.164±6.044a** | **22.498±8.939b** |
| +LTS | 0 | -5.794±4.612 | **28.612±8.781a** | -5.794±4.612 | **28.612±8.781a** |
|  | 20 | **-10.628±2.679** | **64.648±11.348b** | 4.350±3.888 | **78.078±7.477b** |
|  | 40 | **-12.676±1.070** | **83.665±8.107c** | 1.853±2.914 | **88.978±13.281b** |
|  | 60 | 1.953±1.584 | **59.887±3.935b** | -0.332±8.038 | **77.077±18.380b** |

**Table S4** Recovery of the total free amino acids (FAA), Argine (Arg) and Protine (Pro) in *Pogonatum cirratum* subsp. *fuscatum* and *Hypnum plumaeforme* exposed to the indicated amount of N addition, with or without low temperature stress after a 10-day-recovery period (+LTS and –LTS respectively). Data are presented as means ± S.D. (n=3). Different letters indicate significant differences between samples exposed to various levels of N addition under each temperature treatment (*p* < 0.05, one-way ANOVA, LSD test). Data in bold format represent significant differences with 0 (*p* < 0.05, t-test).

| N treatment  (kg N hm^-2^ yr^-1^) | | Nitrate treatment | | | Ammonium treatment | | |
| --- | --- | --- | --- | --- | --- | --- | --- |
|  |  | FAA  (μmol g^-1^ Fw) | Arg  (μmol g^-1^ Fw) | Pro  (μmol g^-1^ Fw) | FAA  (μmol g^-1^ Fw) | Arg  (μmol g^-1^ Fw) | Pro  (μmol g^-1^ Fw) |
| ***Pogonatum cirratum* subsp. *fuscatum*** | | | | | | |  |
| -LTS | 0 | -0.530±0.953a | -0.019±0.059 | -0.020±0.013 | -0.530±0.953a | -0.019±0.059 | -0.020±0.013a |
|  | 20 | 0.824±1.671a | -0.071±0.139 | -0.005±0.018 | **4.433±1.573b** | -0.551±0.150 | 0.036±0.018b |
|  | 40 | **7.690±2.858b** | 0.218±0.042 | -0.011±0.013 | 4.304±2.817b | -1.577±0.139 | 0.037±0.030b |
|  | 60 | -1.103±0.615a | 0.052±0.159 | -0.026±0.021 | 1.419±2.402ab | -2.206±1.117 | 0.012±0.012ab |
| +LTS | 0 | **-3.694±1.163c** | **-0.102±0.014** | -0.010±0.013 | **-3.694±1.163c** | **-0.102±0.014** | -0.010±0.013c |
|  | 20 | -3.154±2.126c | -0.070±0.034 | 0.003±0.010 | **-14.672±0.637b** | 0.272±0.256 | -0.037±0.021c |
|  | 40 | **-7.432±0.389b** | -0.006±0.058 | 0.003±0.007 | **-16.671±2.377b** | -0.187±0.952 | **-0.086±0.025b** |
|  | 60 | **-13.005±1.310a** | -0.055±0.080 | -0.024±0.022 | **-29.603±2.131a** | -1.392±0.619 | **-0.121±0.005a** |
| ***Hypnum plumaeforme*** | | | | | | |  |
| -LTS | 0 | **0.686±0.210c** | 0.004±0.157 | 0.010±0.009c | **0.686±0.210** | 0.004±0.157 | 0.010±0.009 |
|  | 20 | **-9.793±0.376b** | -0.269±0.111 | **-0.051±0.017a** | -1.373±3.554 | 0.050±1.029 | -0.006±0.004 |
|  | 40 | **-11.124±1.615b** | -0.091±0.377 | -0.002±0.004bc | -0.810±2.373 | -0.360±0.646 | 0.017±0.010 |
|  | 60 | **-19.980±1.658a** | 0.093±0.223 | -0.032±0.028ab | -1.227±2.076 | **-1.591±0.572** | 0.011±0.011 |
| +LTS | 0 | -5.099±2.107b | -0.255±0.112 | -0.020±0.016b | -5.099±2.107 | -0.255±0.112 | -0.020±0.016 |
|  | 20 | -3.502±2.118b | -0.328±0.135 | **-0.057±0.006a** | **-5.464±0.991** | 0.192±0.552 | **-0.055±0.020** |
|  | 40 | **-12.499±2.868a** | -0.491±0.192 | **-0.064±0.011a** | **-8.805±1.113** | 0.350±0.406 | -0.044±0.030 |
|  | 60 | **-15.210±2.073a** | -0.627±0.528 | **-0.064±0.016a** | **-7.203±2.096** | **0.366±0.082** | -0.055±0.031 |

**Table S5** Contents of soluble sugar (TSS) and starch in *Pogonatum cirratum* subsp. *fuscatum* and *Hypnum plumaeforme* exposed to the indicated amount of N addition, with or without low temperature stress after a 10-day-recovery period (+LTS and –LTS respectively). Data are presented as means ± S.D. (n=3). Different letters indicate significant differences between samples exposed to various levels of N addition under each temperature treatment (*p* < 0.05, one-way ANOVA, LSD test). Data in bold format represent significant differences with 0 (*p* < 0.05, t-test)..

| N treatment  (kg N ha^-1^) | | Nitrate teatment | | Ammonium treatment | |
| --- | --- | --- | --- | --- | --- |
|  |  | TSS  (mg g^-1^Fw) | Starch  (mg g^-1^Fw) | TSS  (mg g^-1^Fw) | Starch  (mg g^-1^Fw) |
| ***Pogonatum cirratum* subsp. *fuscatum*** | | | | | |
| -LTS | 0 | 19.00±0.85 a | 36.06±0.29 c | 19.00±0.85 a | 36.06±0.29 b |
|  | 20 | 21.35±0.94 b | 36.50±1.31 c | 21.35±2.87 a | 35.88±0.15 b |
|  | 40 | 20.71±0.55 ab | 31.84±1.45 b | 21.42±1.52 a | 33.14±0.83 a |
|  | 60 | 20.70±1.32 ab | 28.07±1.09 a | 19.12±5.71 a | 31.53±0.77 a |
| +LTS | 0 | 21.84±1.19 a | **28.08±1.67 b** | 21.84±1.19 a | **28.08±1.67 a** |
|  | 20 | 19.26±1.99 a | **28.37±1.12 b** | 23.15±1.18 ab | **27.99±1.26 a** |
|  | 40 | 19.54±1.64 a | **26.36±0.68 a** | **25.10±1.31 b** | **26.68±0.47 a** |
|  | 60 | 20.28±0.84 a | **25.51±0.34 a** | 23.66±2.05 ab | **25.78±1.52 a** |
| R.-LTS | 0 | 18.59±1.10 b | 34.04±1.07 b | 18.59±1.10 a | 34.04±1.07 c |
|  | 20 | 17.92±1.07 b | 34.81±0.75 b | 18.67±1.14 a | 31.27±0.47 b |
|  | 40 | 15.87±0.31 a | 28.46±0.88 a | 17.50±0.52 a | 29.27±0.66 a |
|  | 60 | 14.87±0.36 a | 27.11±1.02 a | 17.12±1.98 a | 29.27±0.37 a |
| R.+LTS | 0 | 19.96±0.94 a | **31.32±0.05 b** | 19.96±0.94 a | **31.32±0.05 b** |
|  | 20 | 19.03±0.66 a | **27.28±0.96 a** | 19.64±0.59 a | 32.72±0.65 b |
|  | 40 | **22.61±0.57 b** | 30.90±2.38 ab | **20.03±0.71 a** | 31.81±0.70 b |
|  | 60 | **22.96±0.53 b** | 27.51±0.20 a | **24.46±1.71 b** | 28.94±0.62 a |
| ***Hypnum plumaeforme*** | | | | | |
| -LTS | 0 | 14.28±0.89 a | 14.28±1.18 a | 14.28±0.89 a | 14.28±1.18 a |
|  | 20 | 13.56±0.31 a | 15.80±1.49 ab | 14.19±0.22 a | 14.06±0.70 a |
|  | 40 | 14.04±1.07 a | 17.15±0.23 b | 14.62±1.02 a | 16.06±0.68 b |
|  | 60 | 15.42±0.40 a | 17.19±0.87 b | 16.38±1.04 a | 18.15±0.80 b |
| +LTS | 0 | 15.98±0.48 a | 14.22±1.07 a | 15.98±0.48 a | 14.22±1.07 a |
|  | 20 | **17.59±1.03 a** | 14.71±0.47 a | **23.47±0.88 b** | 13.55±0.13 a |
|  | 40 | **17.77±1.55 a** | **13.80±1.67 a** | **23.52±1.24 b** | **14.97±0.28 b** |
|  | 60 | **21.23±0.59 b** | **14.26±0.62 a** | **21.39±1.93 b** | **13.64±1.09 a** |
| R.-LTS | 0 | 14.43±1.30 a | 14.48±0.72 a | 14.43±1.30 a | 14.48±0.72 a |
|  | 20 | 14.42±1.11 a | 17.66±0.86 b | 18.90±0.63 b | 20.15±0.78 b |
|  | 40 | 17.99±0.98 b | 18.44±0.73 b | 20.01±2.51 ab | 19.05±0.47 b |
|  | 60 | 17.78±0.54 a | 16.71±0.90 b | 22.18±2.37 b | 18.63±1.63 b |
| R.+LTS | 0 | **22.29±0.51 a** | 14.60±0.73 a | **22.29±0.51 a** | 14.60±0.73 a |
|  | 20 | **22.41±1.92 a** | **15.78±0.53 a** | 21.71±1.69 a | **16.88±0.80 b** |
|  | 40 | **20.80±1.38 a** | 17.99±1.09 b | 22.62±2.01 a | 17.64±1.61 c |
|  | 60 | **21.84±2.48 a** | **14.62±0.82 c** | **27.76±1.30 b** | **13.88±0.20 d** |
