# Supplementary material for: Nitrogen Addition Exacerbates the Negative Effects of Low Temperature Stress on Carbon and Nitrogen Metabolism in Moss
Source: Front Plant Sci. 2017 Aug 2;8:1328. doi: 10.3389/fpls.2017.01328 (PMC5539086; doi:10.3389/fpls.2017.01328)
Supplement: Supplementary file 2 [file Image1.PDF]

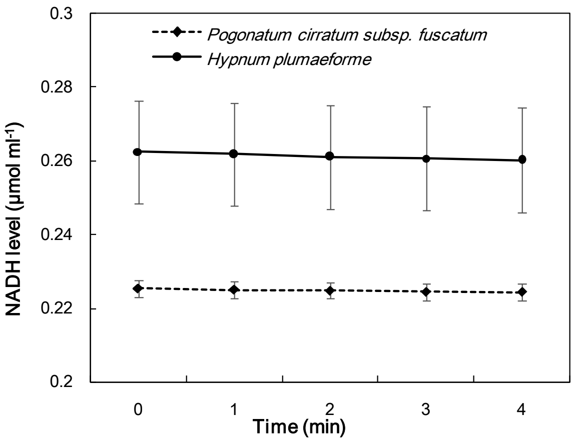

Fig.S1 Change of NADH in RuBPC reaction system in absence of RuBP, creatine, ATP, phosphocreatine kinase, 3-phosphoglycerate kinase and glyceraldehyde-3-phosphate dehydrogenase. Data are presented as means ± S.D. (n=3).

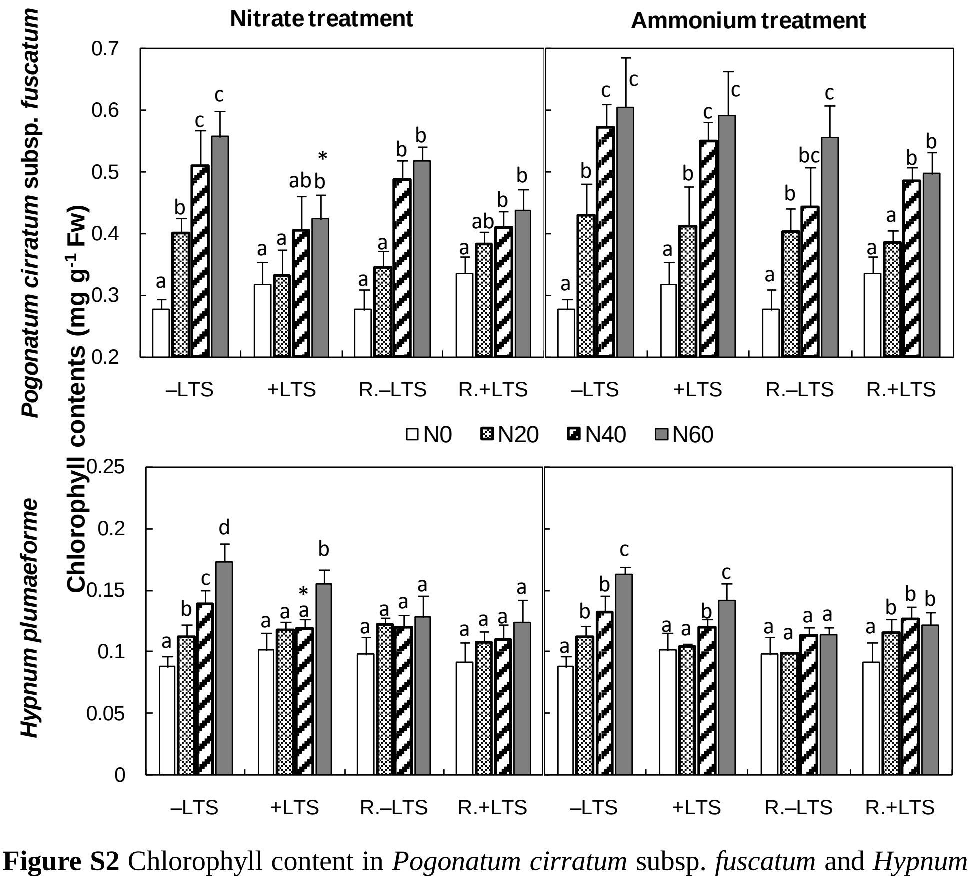

Figure S2 Chlorophyll content in *Pogonatum cirratum* subsp. *fuscatum* and *Hypnum plumaeforme* after N addition (–LTS) and low temperature stress (+LTS) treatments, and after 10-day-recovery from the treatments (R.–LTS and R.+LTS, respectively). Data are presented as means + S.D. (n=3). Different letters on the bars indicate significant differences between samples exposed to various N concentrations under each temperature treatment ($p < 0.05$, one-way ANOVA, LSD test). * on the bars in the +LTS and R.+LTS groups indicate significant differences between corresponding +LTS and –LTS, and R.+LTS and R.–LTS data, respectively ($p < 0.05$, t-test).

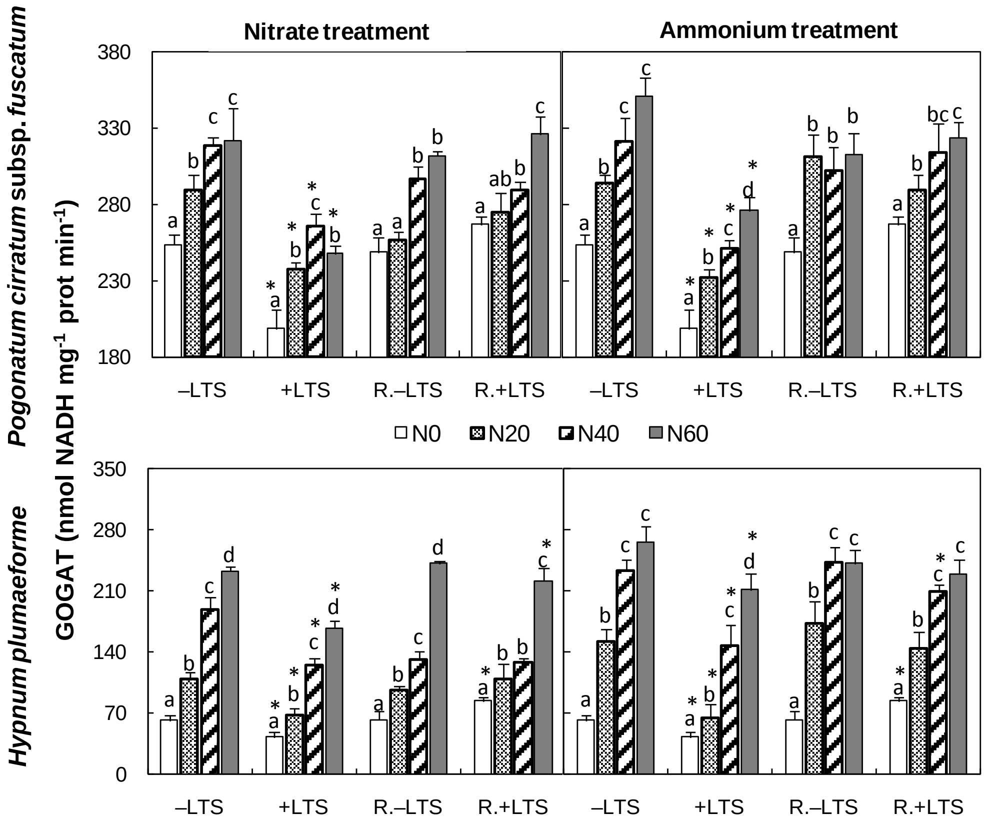

Figure S3 Glutamate synthase (GOGAT) activity in *Pogonatum cirratum* subsp. *fuscatum* and *Hypnum plumaeforme* after N addition (–LTS) and low temperature stress (+LTS) treatments, and after 10-day-recovery from the treatments (R.–LTS and R.+LTS, respectively). Data are presented as means + S.D. (n=3). Different letters on the bars indicate significant differences between samples exposed to various N concentrations under each temperature treatment ($p < 0.05$, one-way ANOVA, LSD test). * on the bars in the +LTS and R.+LTS groups indicate significant differences between corresponding +LTS and –LTS, and R.+LTS and R.–LTS data, respectively ($p < 0.05$, t-test).
